# Body mass index in young men in Switzerland after the national shutdowns during the COVID-19 pandemic: results from a cross-sectional monitoring study at the population level since 2010

**DOI:** 10.1093/eurpub/ckac111

**Published:** 2022-08-22

**Authors:** Samuel Meili, Marek Brabec, Frank Rühli, Thomas W Buehrer, Nejla Gültekin, Zeno Stanga, Nicole Bender, Kaspar Staub, Emilie Reber

**Affiliations:** Department of Diabetes, Endocrinology, Nutritional Medicine and Metabolism, University Hospital and University of Bern, Bern, Switzerland; Institute of Evolutionary Medicine, University of Zurich, Zurich, Switzerland; Institute of Computer Science of the Czech Academy of Sciences, Prague, Czech Republic; Institute of Evolutionary Medicine, University of Zurich, Zurich, Switzerland; Swiss Armed Forces, Medical Services, Ittigen, Switzerland; Swiss Armed Forces, Medical Services, Ittigen, Switzerland; Centre of Competence for Military and Disaster Medicine, Swiss Armed Forces, Ittigen, Switzerland; Department of Diabetes, Endocrinology, Nutritional Medicine and Metabolism, University Hospital and University of Bern, Bern, Switzerland; Centre of Competence for Military and Disaster Medicine, Swiss Armed Forces, Ittigen, Switzerland; Institute of Evolutionary Medicine, University of Zurich, Zurich, Switzerland; Swiss School of Public Health SSPH+, Zurich, Switzerland; Institute of Evolutionary Medicine, University of Zurich, Zurich, Switzerland; Zurich Center for Integrative Human Physiology (ZIHP), University of Zurich, Zurich, Switzerland; Swiss School of Public Health SSPH+, Zurich, Switzerland; Department of Diabetes, Endocrinology, Nutritional Medicine and Metabolism, University Hospital and University of Bern, Bern, Switzerland; Centre of Competence for Military and Disaster Medicine, Swiss Armed Forces, Ittigen, Switzerland

## Abstract

**Background:**

Owing to the coronavirus disease pandemic, the Swiss goverment imposed a shutdown twice in 2020, which may have changed diet and physical activity. Regarding the question of weight change during the pandemic, little information based on measured weight data is available. We aimed to investigate whether the body mass indices (BMIs) of young Swiss men after the two shutdowns in spring and fall 2020 differed from those of young men examined before the shutdowns.

**Methods:**

We analysed young Swiss men’s BMIs taken during mandatory recruitment for the Swiss Armed Forces at the cross-sectional (not individual longitudinal) monitoring level and across weeks of conscription between January 2010 and July 2021 (*n* = 373 016). These data allow for continuous health monitoring of young men at almost the population level (coverage, >90%). For statistical modelling, we used the generalized additive model (GAM) framework.

**Results:**

We showed that the BMIs of the conscripts examined in the 15 weeks after the two shutdowns in spring and autumn 2020 were not or only slightly different from their baseline values. Sensitivity analyses revealed that this conclusion also holds if the BMI distribution or prevalence of excess weight is assessed. The GAM further showed the significant effects of individual and area-based measures of socioeconomic position and age on BMI.

**Conclusion:**

Our results suggest that lifestyle changes during the pandemic in young men might have been too modest to be reflected in body weight. However, longitudinal data and/or data on women, children, or the elderly may lead to different conclusions.

## Introduction

In the wake of the coronavirus disease (COVID-19) pandemic in spring 2020, the Federal Council decided on the first far-reaching shutdown on 16 March 2020, which was relaxed in several steps between April and June 2020.^1^^,^^2^ Owing to the first national shutdown, the lifestyle of the Swiss population changed markedly. Private and public events were prohibited or restricted. Restaurants, markets and entertainment and leisure facilities such as sport centres, concert halls, libraries, or ski areas were closed, as were schools and universities. Instead of a curfew, the Swiss Federal Council imposed a stay-at-home recommendation.^2^

On the one hand, physical activity behaviour was affected by these restrictions; however, performing outside sport activities was not forbidden.^2^ On the other hand, eating habits, work environments and mental well-being might have also changed, as has been documented in other countries.^3^ These affected lifestyle factors are all well known to be associated with weight gain, overweight and obesity.^4^^,^^5^

In autumn 2020, far-reaching measures were again taken by the Federal Council to limit the peak of the second COVID-19 wave. Spontaneous gatherings of more than 15 people in public areas were prohibited from 19 October 2020. The obligation to wear masks was further extended, and bars, restaurants and clubs were only allowed to serve seated guests.^6^ A distance-learning requirement for higher education institutions was introduced on 2 November 2022, except for public schools, which stayed open. On 11 December 2022, the measures were further strengthened by the Federal Council with a 07:00 pm closing time and closures on Sundays and public holidays.^7^ On 22 December 2020, restaurants, entertainment, leisure and sports facilities across Switzerland were again closed, and these restrictions were only relaxed at the beginning of March 2021.^8^

Nationwide mobility tracking in Switzerland showed a striking reduction in the daily travel distance during both periods.^9^ Younger people were affected in particular, as they are the most mobile group of the population.^9^ At present, how these two shutdown periods affected the health of the general population in Switzerland, especially concerning body weight, is unclear. On the basis of the literature^10–13^ nutritional status could have been influenced in two directions: Increased outdoor activities combined with healthier nutrition (homemade, ‘slow food’) may have lead to less overweight. However, it is also conceivable that body weight is adversely influenced by increased immobility, unhealthier food and psychological stress.

Since 2020, there has been a steady increase in the number of studies addressing this issue using electronic surveys that capture the health and nutritional statuses of individuals based on self-reported information during home confinement. Two recent review articles summarize the evidence of weight gain during shutdowns in spring 2020 based on surveys from various countries. The first systematic review and meta-analysis based on 59 000 people from 32 different countries documents weight gain regardless of age group.^14^ The second review also highlights different clusters of weight change, with people with high baseline body mass indices (BMIs) and low educational levels being particularly affected by weight gain.^15^ For Switzerland, a recent survey based on self-reported weight information showed that between 2019 and 2021, the age group between 45 and 64 years gained more than four times as much weight as the younger age groups.^16^

In general, little information is available on this question of weight change during the pandemic based on measured weight data. Our study intended to change this for Switzerland. We analysed young Swiss men's body measurements taken during mandatory recruitment for the Swiss Armed Forces, which allow for continuous health monitoring of young men at almost the population level. A unique feature of our study is that it circumvents all potential problems with sample selection by investigating almost all conscriptable individuals. At the cross-sectional (not individual longitudinal) monitoring level and across weeks of conscription between January 2010 and July 2021, we investigated whether the BMIs of a group of young Swiss men after the two shutdown periods in spring and fall 2020 differed from those of a group of conscripts examined before the shutdowns. To our knowledge, this is the first such study worldwide based on measured routine weight monitoring data. Comparable data from other countries could validate our results.

## Methods

### Swiss conscription

During conscription for the Swiss Armed Forces, approximately 95% of a birth cohort of men with Swiss citizenship at one time at ages between 18 and 22 years, are examined each year.^17^ A detailed description of the conscription process can be found elsewhere.^17^ Together with other examinations, the medical examination during conscription serves to check the health status and thus the physical fitness for military service of the individual conscript. These examinations enable the monitoring of the health status of young men, with unique broad coverage. The six centres where conscription is conducted based on identical protocols are Payerne, Mels, Monteceneri, Rüti, Sumiswald and Aarau. All measurements were performed by trained army medical personnel or specifically trained soldiers serving in the conscription process. In addition to health data, the current occupation and place of residence were recorded.

In the summer of 2018, the Swiss Armed Forces introduced a more flexible conscription system into the armed forces, in which young men themselves determine the timing of their conscription based on their desired date of basic training occurring approximately 12 months later (we controlled our analyses for the change to this system). Therefore, some young men appear now a bit older at conscription than before; this is why the total numbers of conscripts were lower in 2019 and 2020. However, the sample composition in 2019 and 2020 was similar to those in the years before in terms of age groups, residential region and occupational background (table 1).

**Table 1:** Data overview

Year	2010		2011		2012		2013		2014		2015		2016		2017		2018		2019		2020		2021	
Conscripts	*n*		*n*		*n*		*n*		*n*		*n*		*n*		*n*		*n*		*n*		*n*		*n*	
Total	36792		37251		36272		35673		34914		34724		33785		32201		38188		24517		18869		9830	
**Age groups**	***n***	**%**	***n***	**%**	***n***	**%**	***n***	**%**	***n***	**%**	***n***	**%**	***n***	**%**	***n***	**%**	***n***	**%**	***n***	**%**	***n***	**%**	***n***	**%**
<19.00	8534	23.2	8849	23.8	8890	24.5	8902	25.0	10819	31.0	10832	31.2	10818	32.0	11514	35.8	13327	34.9	8831	36.0	4467	23.7	2306	23.5
19.00–19.99	16751	45.5	17234	46.3	16702	46.0	16413	46.0	15702	45.0	15726	45.3	15479	45.8	13785	42.8	15666	41.0	10208	41.6	8048	42.7	3744	38.1
20.00–20.99	8489	23.1	8298	22.3	7784	21.5	7575	21.2	6170	17.7	6058	17.4	5419	16.0	5212	16.2	6913	18.1	4309	17.6	4609	24.4	2614	26.6
21.00–21.99	3018	8.2	2870	7.7	2896	8.0	2783	7.8	2223	6.4	2108	6.1	2069	6.1	1690	5.2	2282	6.0	1169	4.8	1745	9.2	1166	11.9
Total num	36792	100.0	37251	100.0	36272	100.0	35673	100.0	34914	100.0	34724	100.0	33785	100.0	32201	100.0	38188	100.0	24517	100.0	18869	100.0	9830	100.0
**Grossregionen**	***n***	**%**	***n***	**%**	***n***	**%**	***n***	**%**	***n***	**%**	***n***	**%**	***n***	**%**	***n***	**%**	***n***	**%**	***n***	**%**	***n***	**%**	***n***	**%**
Central Switzerland	4103	11.2	4038	10.8	4072	11.2	3901	10.9	3844	11.0	3792	10.9	3740	11.1	3625	11.3	2917	7.6	2735	11.2	2311	12.2	1064	10.8
Eastern Switzerland	6128	16.7	6218	16.7	5859	16.2	5293	14.8	5293	15.2	5598	16.1	5176	15.3	5073	15.8	4519	11.8	3926	16.0	2963	15.7	1311	13.3
Espace Mittelland	9191	25.0	8867	23.8	8282	22.8	8767	24.6	8524	24.4	8131	23.4	7388	21.9	7269	22.6	12515	32.8	6122	25.0	4550	24.1	2441	24.8
Lake Geneva Region	5362	14.6	5539	14.9	6116	16.9	6359	17.8	5986	17.1	5805	16.7	5795	17.2	5258	16.3	8839	23.1	3393	13.8	2602	13.8	1354	13.8
Northwestern Switzerland	4971	13.5	4972	13.3	4939	13.6	4884	13.7	4936	14.1	4756	13.7	4420	13.1	4463	13.9	3240	8.5	3284	13.4	2480	13.1	1410	14.3
Ticino	1421	3.9	1372	3.7	1377	3.8	1349	3.8	1308	3.7	1323	3.8	1402	4.1	1325	4.1	1344	3.5	1045	4.3	813	4.3	453	4.6
Zurich	5615	15.3	6244	16.8	5627	15.5	5112	14.3	5018	14.4	5304	15.3	5863	17.4	5187	16.1	4811	12.6	4010	16.4	3148	16.7	1796	18.3
Total	36791	100.0	37250	100.0	36272	100.0	35665	100.0	34909	100.0	34709	100.0	33784	100.0	32200	100.0	38185	100.0	24515	100.0	18867	100.0	9829	100.0
**ISEI groups**	***n***	**%**	***n***	**%**	***n***	**%**	***n***	**%**	***n***	**%**	***n***	**%**	***n***	**%**	***n***	**%**	***n***	**%**	***n***	**%**	***n***	**%**	***n***	**%**
Tertile 1 (low)	8428	22.9	8317	22.3	7504	20.7	7321	20.5	7119	20.4	6952	20.0	6591	19.5	6141	19.1	6719	17.6	4942	20.2	3835	20.3	1681	17.1
Tertile 2 (medium)	7998	21.7	7916	21.3	7904	21.8	7745	21.7	6919	19.8	6758	19.5	8963	26.5	8428	26.2	9058	23.7	6439	26.3	5235	27.7	2366	24.1
Tertile 3 (high)	7321	19.9	7658	20.6	7584	20.9	7516	21.1	7341	21.0	7365	21.2	5176	15.3	4905	15.2	5487	14.4	4026	16.4	3260	17.3	1503	15.3
Students	9964	27.1	9740	26.1	9626	26.5	9195	25.8	9508	27.2	9830	28.3	8704	25.8	7438	23.1	8822	23.1	5590	22.8	3565	18.9	2429	24.7
Imprecise	3080	8.4	3620	9.7	3654	10.1	3896	10.9	4027	11.5	3819	11.0	4351	12.9	5289	16.4	8099	21.2	3520	14.4	2974	15.8	1851	18.8
Total	36791	100.0	37251	100.0	36272	100.0	35673	100.0	34914	100.0	34724	100.0	33785	100.0	32201	100.0	38185	100.0	24517	100.0	18869	100.0	9830	100.0
**Mean values**	**m**	**SD**	**m**	**SD**	**m**	**SD**	**m**	**SD**	**m**	**SD**	**m**	**SD**	**m**	**SD**	**m**	**SD**	**m**	**SD**	**m**	**SD**	**m**	**SD**	**m**	**SD**
Height (cm)	178.2	6.5	178.2	6.5	178.3	6.6	178.2	6.6	178.3	6.6	178.4	6.6	178.4	6.6	178.5	6.6	178.5	6.6	178.8	6.6	178.7	6.6	178.7	6.6
Weight (kg)	74.5	13.1	74.5	13.0	74.5	13.1	74.4	13.2	74.3	13.2	74.2	13.3	74.4	13.5	74.3	13.9	74.3	13.8	74.4	13.4	74.6	13.7	74.5	14.0
BMI (kg m^−2^)	23.4	3.8	23.4	3.8	23.4	3.8	23.4	3.8	23.3	3.8	23.3	3.9	23.4	3.9	23.3	4.0	23.3	4.0	23.3	3.9	23.3	4.0	23.3	4.1
**BMI (kg/m^2^) (WHO categories)**	***n***	**%**	***n***	**%**	***n***	**%**	***n***	**%**	***n***	**%**	***n***	**%**	***n***	**%**	***n***	**%**	***n***	**%**	***n***	**%**	***n***	**%**	***n***	**%**
<18.5	1290	3.5	1374	3.7	1402	3.9	1421	4.0	1535	4.4	1640	4.7	1655	4.9	1738	5.4	2060	3.5	1328	3.5	1019	3.5	575	3.5
18.5–24.9	26125	71.0	26409	70.9	25671	70.8	25300	70.9	24805	71.0	24531	70.6	23438	69.4	22313	69.3	25018	71.0	16934	71.0	12944	71.0	6718	71.0
25.0–29.9	7220	19.6	7257	19.5	7077	19.5	6835	19.2	6529	18.7	6445	18.6	6584	19.5	6039	18.8	6687	19.6	4658	19.6	3579	19.6	1828	19.6
≥30.0	2157	5.9	2211	5.9	2122	5.9	2117	5.9	2045	5.9	2108	6.1	2106	6.2	2111	6.6	2324	5.9	1524	5.9	1255	5.9	652	5.9

BMI, body mass index; ISEI, International Socio-Economic Index of Occupational Status.

### Variables

The delivered anonymized data included the age groups of the conscripts (18.00–18.99, 19.00–19.99, 20.00–20.99, 21.00–21.99 and >=22.00 years). To measure body height (cm) and weight (kg) in underwear and barefoot, a stadiometer and regular calibrated scale were used in the conscription centres. BMI [weight (kg)/height (m^2^)] was calculated.

The self-reported occupation of the conscripts is the only variable in the data that indicates socioeconomic background at the individual level. By following established procedures,^18^ the occupations of the conscripts were assigned to the International Socio-Economic Index of Occupational Status (ISEI-08).^19^ The ISEI allows for the comparison of occupations according to their socioeconomic status. The ISEI-08 distribution of the occupations was divided into three tertile groups: low, medium and high. Pupils and students together formed a separate group, as did the conscripts with no or insufficient information about their occupation. The regional attributes [language region (3), larger regions (Grossregionen, 7), MS region (microregional intermediate level for scientific and regional policy purposes, 106) and urbanity] were linked to the data using residential postal code and place of residence before full anonymization and data delivery to the study team. The average Swiss neighbourhood index of socioeconomic position (Swiss-SEP 2.0) was also linked at the municipality level.^20^ Swiss-SEP 2.0 indicates the average socioeconomic situation in a postal code and was developed and made available by the Institute for Social and Preventive Medicine at the University of Bern. For this study, the Swiss-SEP of municipalities was divided into quintiles.

During the course of the COVID-19 pandemic and after national public health interventions, recruitment was suspended in spring and autumn 2020. During the first shutdown, conscription was stopped for 13 weeks, from calendar week 10 (Monday, 9 March 2020)–20 (Friday, 15 May 2020). For the second time, the conscription process was interrupted for 3 weeks, from calendar weeks 44 (Monday, 26 October 2020) to 46 (Friday, 13 November 2020).^21^ In both cases, we describe postshutdown effects as two 15-week stretches, starting in the week of resumption after the shutdowns. This time window was determined before the data analysis. On the one hand, this period of almost 4 months was short enough to track BMI changes at the group level. On the other hand, this time window was also long enough to allow for a sufficiently large sample size and for describing not only short term but also random fluctuations.

### Ethics

The fully anonymized dataset for this retrospective cross-sectional monitoring study was provided by the Armed Forces Medical Services (AFMS) on a contractual basis. The data were exported by the AFMS from the ‘Medizinisches Informationssystem der Armee’ (MEDISA) and were made completely anonymous before release to the study team. As described earlier,^17^ Swiss conscription is mandatory, and the anthropometric measurements used in this study are non-clinical, governmental data. According to the Swiss federal law (‘Bundesgesetz über die militärischen Informationssysteme’ MIG, BG 510.91, Art. 2, 9, 24–29), the Swiss Armed Forces are authorized to make the data accessible for academic research in anonymous form. When dealing with fully anonymized data, no additional ethical approval is needed for analyses based on such governmental data (Swiss Data Privacy Act, SR 235.1; 19.6.1992).

### Statistical analysis

Our analysis was based on the generalized additive model (GAM)^22^^,^^23^ class, using penalized splines for smooth terms to achieve sufficient flexibility and t-distribution (with degrees of freedom estimated from the data) and for error terms to achieve robustness to occasional data coding errors. Further explanations for the choice and advantages of this method are presented in the Supplementary appendix. The structure of the model for the BMI of the *i*th individual of residence municipality *s* at time *t* (coded in weeks since the beginning of the study) allows for the correction of spatial, temporal and individual heterogeneity nuisance effects to extract post-shutdown effects as follows:

$${\mathrm{BMI}}_{\mathrm{ist}}=\beta_{0}+\beta_{\mathrm{urban}}.I\left(\text{municipality}\;s\;\text{is}\;\text{of}\;\text{urban}\;\text{type}\right)+\sum_{k}\alpha_{k}.I\left(\text{individual}\;i\;\text{is}\;\text{of}\;\text{age}\;\text{class}\;k\right)+\sum_{l}\gamma_{l}.I\left(\text{individua}l\;i\;\text{is}\;\text{of}\;\text{ISEI}\;\text{group}\;l\right)+\beta_{\mathrm{scheme}}.I\left(\text{time}\;t\;\text{is}\;\text{before}\;\text{the}\;\text{change}\;\text{in conscription}\;\text{scheme}\right)+s_{\mathrm{year}}\left(\text{year}\;\text{of}\;t\right)+s_{\mathrm{seasonality}}\left(\text{week}\;\text{of}\;t\right)+s_{\mathrm{SEP}}\left(\text{mean}\;\text{SEP}\;\text{value}\;\text{of}\;\text{municipality}\;s\right)+s_{\mathrm{region}}\left(\text{MS}\;\text{region}\;\text{of}\;\text{municipality}\;s\right)+s_{\mathrm{postshutdown}1}\left(\text{week}\;\text{after}\;\text{end}\;\text{of}\;\text{first}\;\text{shutdown}\right){.I\left(t\;\text{is}\;\text{within}\;15\;\text{weeks}\;\text{after}\;\text{the}\;\text{end}\;\text{of}\;\text{fist}\;\text{shutdown}\right)+s_{\mathrm{postshutdown}2}\left(\text{week}\;\text{after}\;\text{end}\;\text{of}\;\text{second}\;\text{shutdown}\right).I\left(t\;\text{is}\;\text{within}\;15\;\text{weeks}\;\text{after}\;\text{the}\;\text{end}\;\text{of}\;\text{second}\;\text{shutdown}\right)+\varepsilon }_{\mathrm{ist}}$$

where

-
$I\left(\cdot \right)$ is an indicator function (assumes a value of 1 if its argument is true; otherwise, a value of 0);
-
$\beta_{0}$ , $\beta_{\mathrm{urban}},\;\beta_{\mathrm{scheme}}\;$are unknown coefficients;
-
$\alpha_{k}$ s and $\gamma_{l}$s are unknown coefficients corresponding to the analysis of variance-like submodels of age and ISEI factors (with usual baseline identifiability restrictions);
-
$s_{\mathrm{year}}$ , $s_{\mathrm{SEP}}$ are smooth terms implemented as cubic splines;
-
$s_{\mathrm{seasonality}}$ has to satisfy (as with any proper seasonal profile) periodic conditions so that it can be implemented as a cyclic cubic spline;
-
$s_{\mathrm{region}}$ is the spatial random effect modelled as the Gaussian Markov random field structure^24^;
-
$\varepsilon_{\mathrm{ist}}∼t\left(0,\nu ,\sigma^{2}\right)$ is a *t*-distributed error term with scale and degrees of freedom parameters estimated from the data. Use of a *t*-distributed error term with rather low degrees of freedom (estimated from data) instead of traditional Gaussian errors makes the model highly robust, that is resistant to potential outliers that might occur in big data that we model;
-
$s_{\mathrm{postlockdown}1}$ and $s_{\mathrm{postlockdown}2}$ are smooth effects of main interest. They are implemented as cubic splines with the restriction $s_{\mathrm{postlockdown}1}\left(0\right)=s_{\mathrm{postlockdown}2}\left(0\right)=0$.

All unknown coefficients and smooth terms were estimated from data through optimization of penalized likelihood, with penalty coefficients estimated using restricted maximum likelihood. The calculations were performed in the R software^25^ with the help of the mgcv library.^22^ Our GAM-based, semiparametric approach is a substantial generalization as compared with the linear or other pre-defined functional shape analysis promoted in traditional approaches such as interrupted time series regression.^26^ In fact, the approach we used allows for non-linear and non-constant effects, as salient effects were important in our analysis of post-shutdown transient effects of *a priori* unknown functional shape.

As a sensitivity check, we used an ordinal regression model with World Health Organization (WHO) BMI categories and a similar structure of explanatory variables to check the results in an alternative and even more robust (or outlier-resistant) way.

## Results

The originally delivered data set consisted of 399 346 individuals. We excluded 4798 conscripts (1.2%) who were marked as ‘non-regular conscripts’, 1279 female conscripts (0.3%) who had volunteered for military service and 20 253 conscripts (5.1%) aged >22 years (because these were special cases where recruitment was severely delayed for various reasons). The data set used for this analysis included 373 016 conscripts examined between 1 January 2010 and 31 July 2021 (93.4% of the delivered data set). No extreme or unreliable values in height or weight had to be excluded. The relative frequency of age groups, greater regions and occupational groups did not markedly change over time (table 1). In 2020 (the most recent full calendar year included in the data set), the mean BMI was 23.30 kg m^−2^ (SD 4.0), 19.6% of the conscripts were overweight and 5.9% were obese. For the first 6 months of 2021, for which data were already available, the BMIs of young men did not change significantly compared with the BMIs in 2020. Overall, the mean BMI and prevalence rates of overweight and obesity have stabilized at a high level since 2010 (table 1).

**Figure 1:**
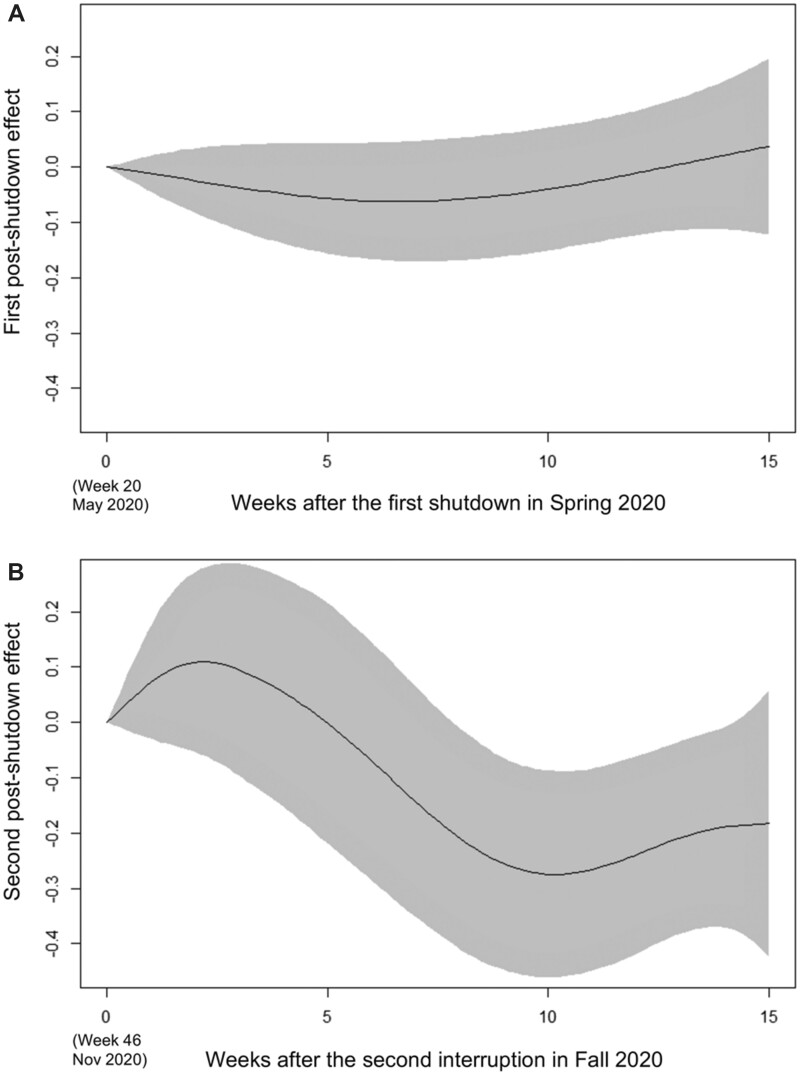
Population-level changes in young men's body mass index (BMI) after the first (A, spring 2020) and second (B, autumn 2020) shutdown ($s_{\mathrm{postshutdown}1}$ and $s_{\mathrm{postshutdown}2}$ model terms). The *y*-axis indicates the effect (partial changes in BMI after correction for seasonality and all other co-factors). The *x*-axis indicates the time in weeks after the end of the shutdowns/interruptions (A: week 0 indicates calendar week 20 in May 2020; B: week 0 indicates calendar week 46 in November 2020). Upper and lower limits of the grey area correspond to the (pointwise constructed) 95% asymptotic intervals.

Our best-fitting GAM (7.7% deviance explained) included two separate post-shutdown effects of 15 weeks each, starting at the end of the shutdowns, and included smoothed terms (for full model results, see Supplementary appendix chapter 1). Figure 1 shows that after the first shutdown in spring 2020, the BMIs of the conscripts entering conscription 15 weeks after the temporary suspension of conscription during the shutdown (and after correction for seasonality and all other co-factors) were not significantly different from the BMIs of the conscripts before the shutdown (figure 1A). However, there was a significant effect (*P* = 0.011) after the second, shorter shutdown in fall 2020 (figure 1B), with a slight increase in BMI in the first 2 weeks, followed by a decrease in BMI until week 15 of the post-shutdown effect. In both cases in figure 1, the confidence intervals are relatively wide, indicating a rather weaker effect.

Our main model also revealed significant differences in BMI for the other included co-variables (see the Supplementary appendix for detailed results). Compared with the reference group of 19-year-old conscripts, older conscripts had significantly higher BMIs, and conscripts in the higher ISEI group and students had significantly lower BMIs. We also found that conscripts living in urban and lower Swiss-SEP areas of Switzerland had higher BMIs (with an almost linear smoothed effect; see Supplementary appendix 1.1, figure B). We also found a significant spatial effect across the 106 MS regions in Switzerland, with the northwestern part of Switzerland having higher BMI values. We also found weak but significant effects for seasonality (lowest BMI values in the summer months) and across years, when the mean BMI of the Swiss conscripts have slightly decreased since 2010.

The same conclusion (only weak effects of shutdown periods on BMI) could be drawn when the model was stratified by larger regions or when we used the heteroskedastic GAMLSS (generalized additive models for location, scale and shape) as an alternative (considering the nature of the BMI distribution). The conclusion also did not change when ordinal regression models were used to assess the WHO categories for BMI.

## Discussion

In this study, we analysed whether shutdowns during the COVID-19 pandemic affected the measured body weights of young men in Switzerland. We showed that the BMIs of the conscripts who were examined during the 15 weeks after the two shutdowns in spring and autumn 2020 were not or only slightly different from the baseline values. The sensitivity analyses revealed that this conclusion also holds if the BMI distribution or prevalence rates of overweight and/or obesity are assessed. The models further showed strong effects of individual and area-based SEPs and age on BMI.

As the number of similar studies based on measured monitoring data is still small, contextualization with the literature is currently difficult. Comparison with many survey studies based on self-reported before–after weight data is challenging in many aspects. Many of these survey studies have reported weight gain during the pandemic, especially for women, previously overweight people and older people.^27^ This was also recently shown by the only Swiss study on this topic.^16^ The reasons for the young men in our data not gaining weight might be multifactorial. One possible explanation could be that the shutdowns in Switzerland were relatively unrestrictive. Despite the recommendation to stay at home, the Swiss population was allowed to go outside at any time. Even with the visible effect of the shutdowns in national mobility tracking,^9^ the more local movement patterns of young people might not have been restricted. Another possibility is that changes in the balance between diet and exercise level over several weeks are less likely to translate into additional body weight in younger people than in older people. One possible explanation could be that lipid turnover in fat tissue decreases during ageing.^28^ In future events such as shutdowns, health monitoring of young men should include not only anthropometric parameters but also body composition and metabolic parameters, which are particularly relevant in terms of later health risks.

Early in the pandemic, overweight and/or obesity were found to be associated with an increased risk of severe infection occurring with COVID-19.^29^ This is also known for influenza and Swine flu, the last pandemic before COVID-19.^30^ Monitoring population weight changes during pandemics is important because, as described earlier, overweight or obesity is an increased risk factor of disease severity, and a considerable number of the Swiss population carry this risk factor.^31^ The immune system plays a key role in the inflammation of adipose tissue caused by obesity and in the pathogenesis of COVID-19.^32^ Furthermore, home office and emotional stress have been shown to be associated with weight gain in already overweight study participants and increased eating when bored and eating when stressed during the COVID-19 pandemic.^33^ Overweight study participants also reported increased stress and loneliness during the COVID-19 pandemic, which could be further exacerbated in a vicious cycle.^34^ To counteract weight gain, it is important to promote behaviours such as staying physically active, maintaining a healthy and planned diet and reducing emotional eating at the population level, especially also during a longer-lasting pandemic.^33^ Specifically, the psychosocial well-being of overweight people should be given continuous attention during, but also afterwards, an ongoing pandemic to counteract an increase in the incidence rates of non-communicable diseases and poor pandemic outcome in a timely manner.^34^

Our study had several limitations. First, we considered only young Swiss men in our study. Women were not considered even though they are particularly at risk of gaining weight according to a French study.^11^ A second limitation is that we only used cross-sectional data. To make a statement about the weight profile of an individual, it would be necessary to collect longitudinal, individual measurements before and after the pandemic. Currently, such data exist only in the form of self-reported weight information from online-surveys. Furthermore, we have no information on how the lifestyles of young men have changed during shutdowns, especially regarding diet, physical activity, psychological condition and well-being. However, this information would be crucial for putting the observed effect in a broader context. Lastly, the use of BMI is a limitation, as it only reflects the relationship between weight and height and does not provide information about body composition. Therefore, changes in body composition and/or metabolic parameters might also have occurred in the young men in this study during and after the shutdowns, which, however, were not reflected in additional body weight.

Prevention is the most effective therapy.^28^^,^^35^ It is important to understand how the behaviour and health of a population adapt and change in response to a crisis on a larger scale. Surveillance and monitoring of the population and public health are the basic prerequisites for possible targeted interventions and are therefore of paramount importance. This especially applies to young men because being obese in adolescence increases the risk of being obese as an adult, and the disease risks associated with excess weight in men in particular increase with age.^28^ Certainly, it would be wrong to say that no prevention against weight gain is needed in the event of another shutdown among young men, based on our study results. For successful prevention, not only should risk factors be addressed but also underlying risk-reducing resources should be identified and strengthened. For this purpose, the results obtained from our study population could also be useful for further studies in other population groups (women, children and elderly people) not only in Switzerland but also in other countries.

## Supplementary data

Supplementary data are available at *EURPUB* online.

## Supplementary Material

ckac111_Supplementary_Data

## Data Availability

This article used fully anonymized individual data as provided by the Swiss Armed Forces upon signing a data contract. By this contract, the authors of the article are not authorized to make individual data sets publicly available. However, other researchers may request the data set from the Swiss Armed Forces by submitting a study protocol. The R-code for the analysis of the data sets can be obtained from the corresponding author.
