# Supplementary material for: Body mass index in young men in Switzerland after the national shutdowns during the COVID-19 pandemic: results from a cross-sectional monitoring study at the population level since 2010
Source: Eur J Public Health. 2022 Aug 22;32(6):955–61. doi: 10.1093/eurpub/ckac111 (PMC9452117; doi:10.1093/eurpub/ckac111)
Supplement: ckac111_Supplementary_Data [file ckac111_supplementary_data.pdf]

Appendix Material to:

Body mass index in young men in Switzerland after the national shutdowns during the COVID-19 pandemic: Results from a cross-sectional monitoring study at the population level since 2010.

Samuel Meili, Marek Brabec, Frank Rühli, Thomas W. Buehrer, Nejla Gültekin, Zeno Stanga, Nicole Bender, Kaspar Staub, Emilie Reber

---

Outline:

1 Statistical Methods 2

2 Full results of the main model 4

2.1 Visualisation of the smoothed terms and MS region effect on BMI 6

2.2 Data-plot of the mean BMI during 2020-2021 8

3 Results from alternative models 9

3.1 Ordinal regression 9

3.2 Heteroscedastic normal regression 11

1 Statistical Methods

Our analysis was based on slightly generalised GAM[22,23] class, using penalised splines for smooth terms to achieve sufficient flexibility and t-distribution (with a degree of freedom estimated from data) for the error terms to achieve robustness to occasional data coding errors. There are several important motivations for using the GAM-based approach in this study. First, we investigated several effects, some of which are of direct interest (e.g., the post-shutdown effect) and others that are of secondary interest or that are used for adjustment for nuisance variables and to achieve straightforward interpretability of primary variables. To this end, we need to heavily utilise the structured (modular, additive) nature of the model. In this way, we can address, analyse, test, and discuss different components in a single model.

Next, since we are dealing with effects whose analytical shapes are not known a priori, it is important to allow for flexible treatment (not too restrictive, e.g., a linear or low-degree polynomial class that can easily bias the results). Here, we used a flexible, nonparametric, penalised spline approach. Next, we used a slight generalisation of the original GAM class to include the t-distribution of errors. As the degrees of freedom for this distribution were estimated from the data and turned out to be rather low, it is an important robustness feature. Thus, the model is highly resistant to occasional gross errors that might occur in the data (as they are obtained from careful but routine measurements and not from a specialised laboratory study). Also, the general GAM approach allowed us to treat non-trivial and non-stationary spatial correlation structures in an elegant and computationally effective way using the Gaussian Markov random field structure.[24] The structure of the model for the BMI of the i-th individual of residence municipality s at time t (coded in weeks since the beginning of the study) corrects for spatial, temporal, and individual heterogeneity nuisance effects to extract post-shutdown effects as follows:

BMI_{ist}

$= \beta_0 + \beta_{urban}.I(\text{municipality } s \text{ is of urban type}) + \sum_k \alpha_k.I(\text{individual } i \text{ is of age class } k)$

$+ \sum_l \gamma_l.I(\text{individual } i \text{ is of ISEI group } l)$

$+ \beta_{scheme}.I(\text{time } t \text{is before the change in conscription scheme}) + s_{year}(\text{year of } t)$

$+ s_{seasonality}(\text{week of } t) + s_{SEP}(\text{mean SEP value of municipality } s)$

$+ s_{region}(\text{MS region of municipality } s)$

$+ s_{postshutdown1}(\text{week after end of first shutdown}).I(t \text{ is within 15 weeks after the end of fist shutdown})$

$+ s_{postshutdown2}(\text{week after end of second shutdown}).I(t \text{ is within 15 weeks after the end of second shutdown})$

$+ \varepsilon_{ist}$

where

- $I(.)$ is an indicator function (assumes a value of 1 if its argument is true; otherwise, a value of 0);
- $\beta_0, \beta_{urban}, \beta_{scheme}$ are unknown coefficients;
- α_k's and γ_l's are unknown coefficients corresponding to the analysis of variance (ANOVA)-like submodels of age and ISEI factors (with usual baseline identifiability restrictions);
- s_{year}, s_{SEP} are smooth terms implemented as cubic splines;
- $s_{seasonality}$ has to satisfy (as with any proper seasonal profile) periodic conditions so that it can be implemented as a cyclic cubic spline;
- s_{region} is the spatial random effect modelled as the Gaussian Markov random field structure;[24]
- $\varepsilon_{ist} \sim t(0, \nu, \sigma^2)$ is a t-distributed error term with scale and degrees of freedom parameters estimated from the data. Use of a t-distributed error term with rather low degrees of freedom (estimated from data) instead of traditional Gaussian errors makes the model highly robust, that is, resistant to potential outliers that might occur in big data that we model;

$s_{postlockdown1}$ and $s_{postlockdown2}$ are smooth effects of main interest. They are implemented as cubic splines with the restrictions$s_{postlockdown1}(0) = s_{postlockdown2}(0) =$

2 Full results of the main model

```
Family: Scaled t(3.735,2.679)
Link function: identity

Formula:
BMI ~ s(Year, bs = "cr") + fUrbanNon. Urban + fAgegroup +
fscheme + fISEIGroups + s(Mean_SSEP, bs = "cr") + s(Week_Mon,
bs = "cc") + s(fMSRegion, bs = "mrf", xt = x. MSRegion) +
s(postlockdown1, by = zero, bs = "cr", pc = 0) + s(postlockdown2,
by = zero, bs = "cr", pc = 0)

Parametric Terms:
                df       F  p-value
fUrbanNon.Urban  1  24.327 8.13e-07
fAgegroup        3 756.106  < 2e-16
fscheme          1   0.003    0.957
fISEIGroups      4 434.268  < 2e-16
---
Approximate significance of smooth terms:
                          edf  Ref.df       F  p-value
s(Year)                 5.127   6.228  30.740  < 2e-16
s(Mean_SSEP)            3.966   4.913 133.142  < 2e-16
s(Week_Mon)             6.093   8.000   9.554 2.51e-16
s(fMSRegion)           91.078 101.507  21.928  < 2e-16
s(postlockdown1):zero   1.750   2.136   0.744   0.4679
s(postlockdown2):zero   3.240   3.948   3.459   0.0108
> summary(a.cs.2)

Family: Scaled t(3.735,2.679)
Link function: identity

Formula:
```

```
BMI ~ s(Year, bs = "cr") + fUrbanNon. Urban + fAgegroup +
fscheme + fISEIGroups + s(Mean_SSEP, bs = "cr") + s(Week_Mon,
bs = "cc") + s(fMSRegion, bs = "mrf", xt = x. MSRegion) +
s(postlockdown1, by = zero, bs = "cr", pc = 0) + s(postlockdown2,
by = zero, bs = "cr", pc = 0)

Parametric coefficients:
                               Estimate Std. Error t value Pr(>|t|)
(Intercept)                   22.914251   0.030166 759.601  < 2e-16 ***
fUrbanNon.UrbanUrban           0.069570   0.014105   4.932 8.13e-07 ***
fAgegroup20.00-20.99           0.318353   0.015070  21.124  < 2e-16 ***
fAgegroup21.00-21.99           0.686759   0.022935  29.944  < 2e-16 ***
fAgegroup<19.00               -0.291254   0.013667 -21.310  < 2e-16 ***
fschemeold                     0.001864   0.034516   0.054    0.957
fISEIGroupsWithout occupation -0.426096   0.039082 -10.903  < 2e-16 ***
fISEIGroupsTertile 1 (low)     0.109151   0.016116   6.773 1.27e-11 ***
fISEIGroupsTertile 3 (high)   -0.145160   0.016511  -8.792  < 2e-16 ***
fISEIGroupsStudents           -0.549195   0.016464 -33.358  < 2e-16 ***

Signif. codes:  0 ‘***’ 0.001 ‘**’ 0.01 ‘*’ 0.05 ‘.’ 0.1 ‘ ’ 1

Approximate significance of smooth terms:
                         edf  Ref.df       F  p-value
s(Year)                5.127   6.228  30.740  < 2e-16 ***
s(Mean_SSEP)           3.966   4.913 133.142  < 2e-16 ***
s(Week_Mon)            6.093   8.000   9.554 2.51e-16 ***
s(fMSRegion)          91.078 101.507  21.928  < 2e-16 ***
s(postlockdown1):zero  1.750   2.136   0.744   0.4679
s(postlockdown2):zero  3.240   3.948   3.459   0.0108 *
---
Signif. codes:  0 ‘***’ 0.001 ‘**’ 0.01 ‘*’ 0.05 ‘.’ 0.1 ‘ ’ 1

R-sq.(adj) =  0.0218   Deviance explained = 7.74%
```

```
fREML = 4.7316e+05  Scale est. = 1         n = 332320
> AIC(a.cs.2)
[1] 1785788
```

2.1 Visualisation of the smoothed terms and MS region effect on BMI

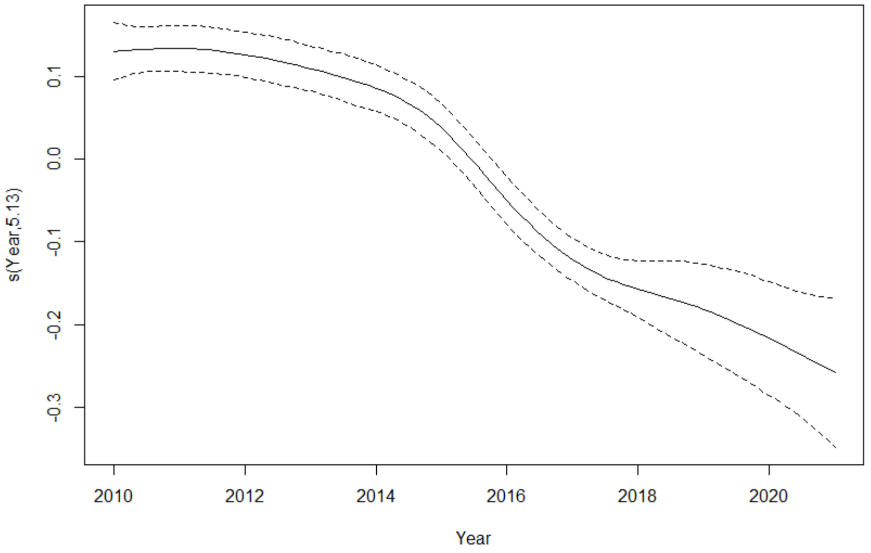

Figure A: Smoothed BMI effect across years

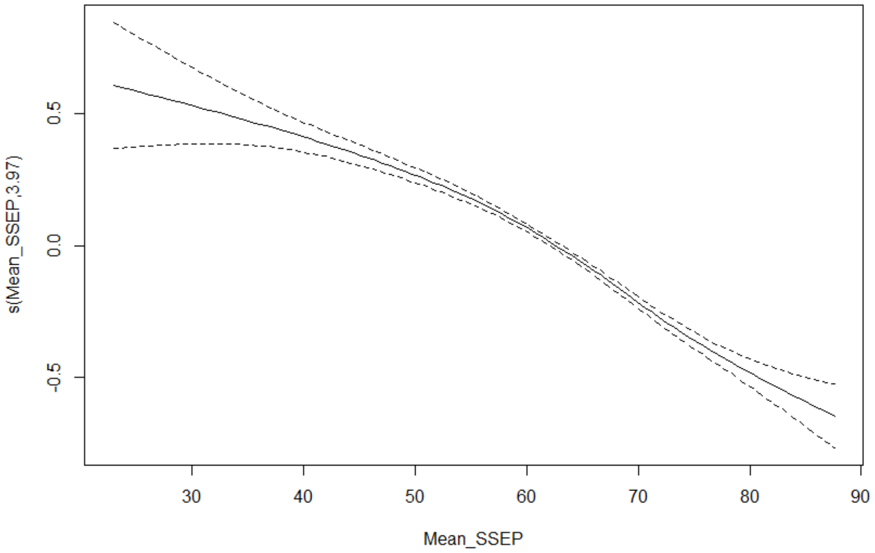

Figure B: Smoothed BMI effect across SSEP

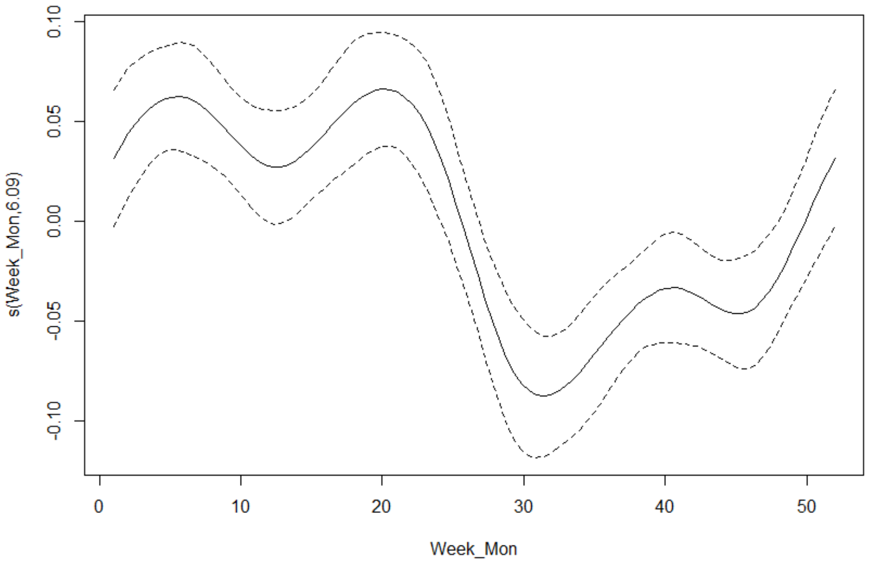

Figure C: Smoothed BMI effect across weeks

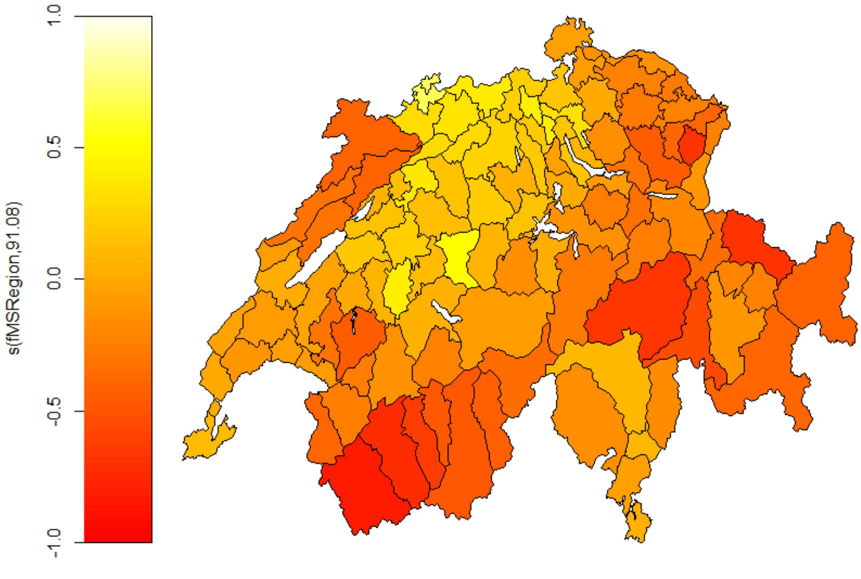

Figure D: Smoothed BMI effect across MS regions

2.2 Data-plot of the mean BMI during 2020-2021

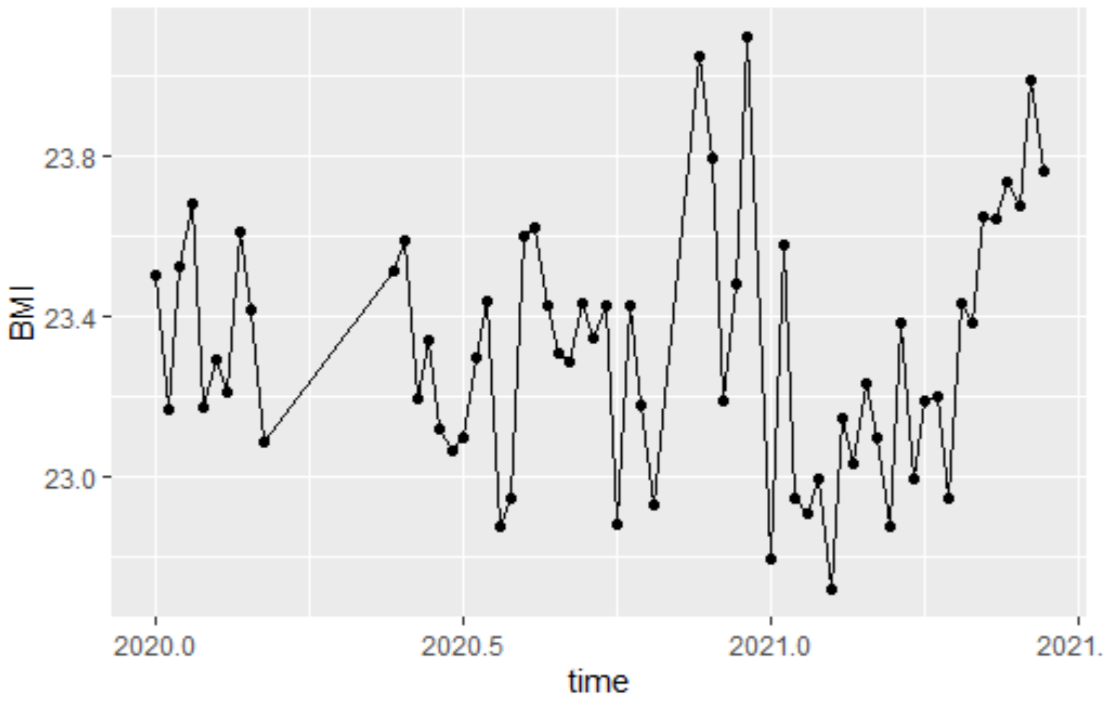

Figure E: Mean BMI of the conscripts during 2020-2021

3 Results from alternative models

3.1 Ordinal regression

Here, we use ordinal regression formulated as a GAM with smooth effects similar to those used previously for BMI modelling. The main motivation is to utilize an alternative model (less influenced by potential outliers or other data problems) as a form of sensitivity check.

```
Family: Ordered Categorical(-1,3.19,4.86,84)
Link function: identity

Formula:
oBMI ~ s(Year, bs = "cr") + fUrbanNon. Urban + fAgegroup +
fscheme + fISEIGroups + s(Mean_SSEP, bs = "cr") + s(Week_Mon,
bs = "cc") + s(fMSRegion, bs = "mrf", xt = x. MSRegion) +
s(postlockdown1, by = zero, bs = "cr", pc = 0) + s(postlockdown2,
by = zero, bs = "cr", pc = 0)

Parametric Terms:
                df       F  p-value
fUrbanNon.Urban  1  27.631 1.47e-07
fAgegroup        3 508.905  < 2e-16
fscheme          1   2.201    0.138
fISEIGroups      4 495.313  < 2e-16

Approximate significance of smooth terms:
                          edf Ref.df       F p-value
s(Year)                 5.174  6.295  13.051 < 2e-16
s(Mean_SSEP)            4.144  5.117 124.832 < 2e-16
s(Week_Mon)             5.278  8.000   7.267 < 2e-16
s(fMSRegion)           87.587 99.966  16.613 < 2e-16
s(postlockdown1):zero  2.839  3.470   2.658 0.05172
s(postlockdown2):zero  1.005  1.017   8.182 0.00406
```

```
> AIC(o2)
[1] 575938.1


> summary(o2)

Family: Ordered Categorical(-1,3.19,4.86,84)
Link function: identity

Formula:
oBMI ~ s(Year, bs = "cr") + fUrbanNon. Urban + fAgegroup +
fscheme + fISEIGroups + s(Mean_SSEP, bs = "cr") + s(Week_Mon,
bs = "cc") + s(fMSRegion, bs = "mrf", xt = x. MSRegion) +
s(postlockdown1, by = zero, bs = "cr", pc = 0) + s(postlockdown2,
by = zero, bs = "cr", pc = 0)

Parametric coefficients:
                              Estimate Std. Error t value Pr(>|t|)
(Intercept)                   2.164111   0.020490 105.619  < 2e-16 ***
fUrbanNon.UrbanUrban          0.050210   0.009552   5.257 1.47e-07 ***
fAgegroup20.00-20.99          0.166459   0.010043  16.575  < 2e-16 ***
fAgegroup21.00-21.99          0.362823   0.014948  24.272  < 2e-16 ***
fAgegroup<19.00              -0.172659   0.009382 -18.404  < 2e-16 ***
fschemeold                    0.034897   0.023520   1.484    0.138
fISEIGroupsWithout occupation -0.267770   0.026995  -9.919  < 2e-16 ***
fISEIGroupsTertile 1 (low)    0.077609   0.010648   7.289 3.13e-13 ***
fISEIGroupsTertile 3 (high)  -0.117048   0.011061 -10.582  < 2e-16 ***
fISEIGroupsStudents          -0.404172   0.011235 -35.975  < 2e-16 ***
---
Signif. codes:  0 ‘***’ 0.001 ‘**’ 0.01 ‘*’ 0.05 ‘.’ 0.1 ‘ ’ 1

Approximate significance of smooth terms:
                        edf Ref.df       F p-value
```

```
s(Year)                 5.174  6.295  13.051 < 2e-16 ***
s(Mean_SSEP)            4.144  5.117 124.832 < 2e-16 ***
s(Week_Mon)             5.278  8.000   7.267 < 2e-16 ***
s(fMSRegion)           87.587 99.966  16.613 < 2e-16 ***
s(postlockdown1):zero  2.839  3.470   2.658 0.05172 .
s(postlockdown2):zero  1.005  1.017   8.182 0.00406 **
---
Signif. codes:  0 ‘***’ 0.001 ‘**’ 0.01 ‘*’ 0.05 ‘.’ 0.1 ‘ ’ 1

Deviance explained = 1.11%
fREML = 7.03e+05  Scale est. = 1         n = 332320
```

3.2 Heteroscedastic normal regression

```
Family: gaulss
Link function: identity logb

Formula:
BMI ~ s(Year, bs = "cr") + fUrbanNon.Urban + fAgegroup +
fscheme + fISEIGroups + s(Mean_SSEP, bs = "cr") + s(Week_Mon,
bs = "cc") + s(fMSRegion, bs = "mrf", xt = x.MSRegion) +
s(postlockdown1, by = zero, bs = "cr", pc = 0) + s(postlockdown2,
by = zero, bs = "cr", pc = 0)
~s(Year, bs = "cr") + fUrbanNon.Urban + fAgegroup + fscheme +
fISEIGroups + s(Mean_SSEP, bs = "cr") + s(Week_Mon,
bs = "cc") + s(fMSRegion, bs = "mrf", xt = x.MSRegion) +
s(postlockdown1, by = zero, bs = "cr", pc = 0) + s(postlockdown2,
by = zero, bs = "cr", pc = 0)

Parametric Terms:
                  df   Chi.sq  p-value
```

```
fUrbanNon.Urban    1   42.884 5.81e-11
fAgegroup          3 2084.781  < 2e-16
fscheme            1    1.523    0.217
fISEIGroups        4 2653.398  < 2e-16
fUrbanNon.Urban.1  1  139.207  < 2e-16
fAgegroup.1        3 1055.304  < 2e-16
fscheme.1          1    2.471    0.116
fISEIGroups.1      4 2532.459  < 2e-16

Approximate significance of smooth terms:
                            edf  Ref.df   Chi.sq p-value
s(Year)                   6.299   7.427  117.003 < 2e-16
s(Mean_SSEP)              4.546   5.522  806.765 < 2e-16
s(Week_Mon)               5.717   8.000   56.932 < 2e-16
s(fMSRegion)             90.552 101.290 2129.421 < 2e-16
s(postlockdown1):zero     2.322   2.820    5.588 0.11021
s(postlockdown2):zero     3.952   4.793   15.897 0.00592
s.1(Year)                 8.609   8.950  144.468 < 2e-16
s.1(Mean_SSEP)            5.011   6.017  882.793 < 2e-16
s.1(Week_Mon)             4.686   8.000   17.579 0.00115
s.1(fMSRegion)           89.891 100.930 1703.167 < 2e-16
s.1(postlockdown1):zero   3.676   4.482   11.458 0.02479
s.1(postlockdown2):zero   3.754   4.563   12.514 0.02156
> summary(h2)

Family: gaulss
Link function: identity logb

Formula:
BMI ~ s(Year, bs = "cr") + fUrbanNon.Urban + fAgegroup +
fscheme + fISEIGroups + s(Mean_SSEP, bs = "cr") + s(Week_Mon,
bs = "cc") + s(fMSRegion, bs = "mrf", xt = x.MSRegion) +
s(postlockdown1, by = zero, bs = "cr", pc = 0) + s(postlockdown2,
```

```
by = zero, bs = "cr", pc = 0)
~s(Year, bs = "cr") + fUrbanNon.Urban + fAgegroup + fscheme +
fISEIGroups + s(Mean_SSEP, bs = "cr") + s(Week_Mon,
bs = "cc") + s(fMSRegion, bs = "mrf", xt = x.MSRegion) +
s(postlockdown1, by = zero, bs = "cr", pc = 0) + s(postlockdown2,
by = zero, bs = "cr", pc = 0)

Parametric coefficients:
                                Estimate Std. Error z value Pr(>|z|)
(Intercept)                    23.489049   0.038207 614.776  < 2e-16 **
*
fUrbanNon.UrbanUrban            0.108642   0.016590   6.549 5.81e-11 **
*
fAgegroup20.00-20.99            0.350243   0.018178  19.267  < 2e-16 **
*
fAgegroup21.00-21.99            0.753080   0.028849  26.104  < 2e-16 **
*
fAgegroup<19.00                -0.355315   0.015528 -22.883  < 2e-16 **
*
fschemeold                      0.054605   0.044244   1.234    0.217
fISEIGroupsWithout occupation  -0.497002   0.046899 -10.597  < 2e-16 **
*
fISEIGroupsTertile 1 (low)      0.200299   0.019995  10.017  < 2e-16 **
*
fISEIGroupsTertile 3 (high)    -0.203014   0.019788 -10.259  < 2e-16 **
*
fISEIGroupsStudents            -0.741893   0.018841 -39.377  < 2e-16 **
*
(Intercept).1                   1.340836   0.007522 178.246  < 2e-16 **
*
fUrbanNon.UrbanUrban.1          0.037137   0.003148  11.799  < 2e-16 **
*
```

```
fAgegroup20.00-20.99.1          0.040068   0.003349  11.963  < 2e-16 **
*
fAgegroup21.00-21.99.1          0.091549   0.005098  17.958  < 2e-16 **
*
fAgegroup<19.00.1              -0.054712   0.003027 -18.073  < 2e-16 **
*
fschemeold.1                    0.013815   0.008789   1.572    0.116
fISEIGroupsWithout occupation.1 -0.061976   0.008594  -7.211 5.54e-13 **
*
fISEIGroupsTertile 1 (low).1    0.041921   0.003581  11.706  < 2e-16 **
*
fISEIGroupsTertile 3 (high).1  -0.025292   0.003661  -6.908 4.90e-12 **
*
fISEIGroupsStudents.1          -0.139698   0.003654 -38.232  < 2e-16 **
*
---
Signif. codes:  0 ‘***’ 0.001 ‘**’ 0.01 ‘*’ 0.05 ‘.’ 0.1 ‘ ’ 1

Approximate significance of smooth terms:
                           edf  Ref.df   Chi.sq p-value
s(Year)                  6.299   7.427  117.003 < 2e-16 ***
s(Mean_SSEP)             4.546   5.522  806.765 < 2e-16 ***
s(Week_Mon)              5.717   8.000   56.932 < 2e-16 ***
s(fMSRegion)            90.552 101.290 2129.421 < 2e-16 ***
s(postlockdown1):zero    2.322   2.820    5.588 0.11021
s(postlockdown2):zero    3.952   4.793   15.897 0.00592 **
s.1(Year)                8.609   8.950  144.468 < 2e-16 ***
s.1(Mean_SSEP)           5.011   6.017  882.793 < 2e-16 ***
s.1(Week_Mon)            4.686   8.000   17.579 0.00115 **
s.1(fMSRegion)          89.891 100.930 1703.167 < 2e-16 ***
s.1(postlockdown1):zero  3.676   4.482   11.458 0.02479 *
s.1(postlockdown2):zero  3.754   4.563   12.514 0.02156 *
---
```

```
Signif. codes:  0 ‘***’ 0.001 ‘**’ 0.01 ‘*’ 0.05 ‘.’ 0.1 ‘ ’ 1

Deviance explained = 2.67%
-REML = 9.1307e+05  Scale est. = 1         n = 332320
> AIC(h2)
[1] 1825621
```
